# Genomic profile of ovarian carcinomas

**DOI:** 10.1186/1471-2407-14-315

**Published:** 2014-05-05

**Authors:** Francesca Micci, Lisbeth Haugom, Vera M Abeler, Ben Davidson, Claes G Tropé, Sverre Heim

**Affiliations:** 1 Section for Cancer Cytogenetics, Institute for Cancer Genetics and Informatics, The Norwegian Radium Hospital, Oslo University Hospital, 0310 Oslo, Norway; 2 Centre for Cancer Biomedicine, University of Oslo, Oslo, Norway; 3 Department of Pathology, The Norwegian Radium Hospital, Oslo University Hospital, Oslo, Norway; 4 Faculty of Medicine, University of Oslo, Oslo, Norway; 5 Department of Gynecology, The Norwegian Radium Hospital, Oslo University Hospital, Oslo, Norway

**Keywords:** Ovarian carcinoma, Karyotyping, HR-CGH

## Abstract

**Background:**

It is known that all tumors studied in sufficient number to draw conclusions show characteristic/specific chromosomal rearrangements, and the identification of these chromosomes and the genes rearranged behind the aberrations may ultimately lead to a tailor-made therapy for each cancer patient. Knowledge about the acquired genomic aberrations of ovarian carcinomas is still unsatisfactory.

**Methods:**

We cytogenetically analyzed 110 new cases of ovarian carcinoma of different histological subtypes using karyotyping of G-banded chromosomes and high-resolution comparative genomic hybridization. We first compared the aberration patterns identified by the two genomic screening techniques using the so-called “classical” pathological classification in which the carcinomas are grouped as tumors of types I and II. We also broke down our findings according to the more “modern” classification which groups the carcinomas in five different categories.

**Results:**

The chromosomal breakpoints identified by karyotyping tended to cluster to 19p/q and to 11q, but no unquestionably recurrent rearrangement could be seen. Common imbalances were scored as gains from 1q, 3q, 7q, and 8q and losses from 17p, 19q, and 22q. Gain of material from 8q23 and losses from 19q and 22q have previously been described at high frequencies in bilateral and borderline ovarian carcinomas. The fact that they were present both in “precursor” lesions, i.e., borderline tumors, as well as in tumors of more advanced stages, i.e., carcinomas, highlights the possibility of an adenoma-carcinoma sequence in ovarian carcinogenesis.

**Conclusion:**

Based on the relatively simple genomic changes we identified in the low-grade serous carcinomas examined (n = 7) and which largely corresponded to the aberration pattern formerly identified in borderline tumors, one can interpret the cytogenetic data as supporting the view that the low-grade carcinomas represent a phenotypically more advanced stage of borderline tumors. Whether transition from low-grade to high-grade carcinoma also occurs, is a question about which the genomic data is still inconclusive.

## Background

Cancer of the ovary represents 30% of all cancer of the female genital organs [1]. Adenocarcinomas make up 75% of all ovarian tumors and 95% of ovarian malignancies [1]. At the same time, surface epithelial tumors of the ovary display a wide variety of histologic types which may reflect pathogenetic variability and/or differences among the cell(s) of origin. One potentially useful way of classifying these neoplasms is to divide them into those that appear to develop from benign and/or atypical proliferative precursor lesions (mucinous, endometrioid, clear cell, and low-grade serous carcinomas, also referred to as type I tumors) and those that appear to develop *de novo* (high-grade serous carcinomas or type II tumors) [2,3]. However, the apparent difference between serous and nonserous tumors in this regard may reflect the rapidity of the adenoma-carcinoma transition rather than any qualitative pathogenetic difference. Specifically, it can be argued that the adenoma-carcinoma sequence for serous tumors is variable but, on average, shorter than that for mucinous, endometrioid, and clear-cell tumors; this would explain the relative uniformity of serous carcinomas and the infrequent presence in them of neoplastic precursor lesions [2]. It is obvious that use of the term “*de novo*” in this context reflects our ignorance about the early events of ovarian carcinogenesis rather than real insight into the tumor origin.

At the same time, pathologists also classify ovarian carcinomas into five main types based on histopathological, immunohistochemical, and molecular genetic findings: high-grade serous carcinomas, endometrioid carcinomas, clear-cell carcinomas, mucinous carcinomas, and low-grade serous carcinomas [4]. The tumors belonging to these categories account for 98% of ovarian carcinomas. They differ group-wise from one another also with regard to epidemiological and genetic risk factors, precursor lesions, patterns of spreading, molecular events during oncogenesis, response to chemotherapy, and prognosis [4].

Many of the more than 400 (at the moment 438) scientifically reported ovarian carcinomas with karyotypically characterized chromosomal aberrations [5] were examined as abdominal effusions, that is at a very late stage in tumor progression, and many were incompletely karyotyped. Knowledge about the chromosomal characteristics of this type of cancer is therefore far from complete.

We here present 110 new cases of ovarian carcinoma of different histologic subtypes (73 high-grade serous carcinomas, seven low-grade serous, 15 endometrioid, eight clear-cell, three mucinous, three mixed endometrioid and mucinous, and one undifferentiated carcinoma) whose genome was characterized by G-banding followed by karyotyping and high-resolution comparative genomic hybridization (HR-CGH). We specifically compared the acquired genomic aberration pattern of the high-grade serous carcinomas with that of the other types in order to address the question if they share common genomic abnormalities and therefore have a common/similar pathogenesis or, alternatively, if the high-grade serous tumors warrant being considered as a wholly distinct entity. We also scrutinized the aberration patterns of the five different subgroups to identify whatever genomic aberrations might distinguish each group or if any are common to two or more ovarian carcinoma types.

## Methods

### Tumors

The material consisted of fresh samples from 110 primary ovarian carcinomas surgically removed at The Norwegian Radium Hospital from 1999 to 2004 (Additional file 1: Table S1). Neoadjuvant therapy had been given in 25 of the cases before surgery (Additional file 1: Table S1). The tumors were classified as high-grade serous adenocarcinoma (73 samples), low-grade serous adenocarcinoma (seven samples), endometrioid carcinoma (15 samples), clear-cell carcinoma (eight samples), mucinous carcinoma (three samples), undifferentiated carcinoma (one sample), and mixed histology between endometroid and mucinous carcinoma (three samples and cases).

The project has been approved by the Regional Ethics Committee for Medical and Health Research Ethics South-East in the year 2007 (project number 2.2007.425). Written informed consent was obtained from the patients. Frozen tissue was retrieved from The Radium Hospital biobank (nr 1971, S-07194a approved by the Regional Ethics Committee and The Directory of Health (Helsedirektorat) in 2007). The entire study was also approved by the institutional review board at the Norwegian Radium Hospital.

### Cell culturing and karyotyping

The tumor samples were manually minced and disaggregated with Collagenase II (Worthington, Freehold, NJ, USA) until a suitable suspension of cells and cell clumps was obtained. After 6-7 days of culturing in a selective medium [6], colchicine was added and the cultures harvested according to Mandahl [7]. The chromosomes of the dividing cells were then G-banded and a karyotype established according to the recommendations of the ISCN [8].

### High-resolution comparative genomic hybridization (HR-CGH)

DNA was isolated by the phenol-chloroform method as previously described [9]. CGH was performed according to our modifications of standard procedures [10,11]. Chromosomes were karyotyped based on their inverted DAPI appearance and the relative hybridization signal intensity was determined along each chromosome. On average, 10-15 metaphases were analyzed. The HR-CGH analysis was performed as previously described [12]. The description of the CGH copy number changes was based on the recommendation of the ISCN [8].

## Results

Karyotypic information was obtained on 86 of the original 110 samples (Additional file 1: Table S1). The 24 cultures that failed showed either no cell attachment after several days *in vitro* or no metaphases. A normal karyotype was found in 22 tumors. The remaining 64 tumors (Additional file 1: Table S1) mostly showed complex karyotypes with several structural and numerical aberrations. Simple karyotypes, by which we mean karyotypes showing less than four aberrations, were seen only in one high-grade serous carcinoma (case 35), two low-grade serous carcinomas (cases 77 and 78), one clear-cell carcinoma (case 102), and one mixed endometrioid and mucinous carcinoma (case 107). Case 35 (a high-grade serous tumor) showed monosomies of chromosomes X, 4, and 10. Case 77 (a low-grade serous tumor) showed two unrelated clones with monosomy 11 and monosomy of the X chromosome, respectively. Case 78 (a low-grade serous tumor) showed trisomy 7 as the sole abnormality in all cells analyzed. Case 102 (a clear-cell tumor) had two supernumerary markers of unknown origin in three metaphases of the 45 cells analyzed. Case 107 (also a mixed endometrioid and mucinous carcinoma) showed additional material of unknown origin on 8q24 as the sole chromosomal abnormality. The only tumor showing obvious clonal evolution (i.e., beyond having several aberrations) was case 38 (a high-grade serous carcinoma) which had three related clones with only numerical aberrations corresponding to the karyotype 47 ~ 49,XX,+8,+9[2]/49,idem,+5,-6,+7[4]/54,idem,+3,+5,+6,+7,+14,+17,+19[5]. Breakpoint clusters were seen at chromosome arms 11q (33 out of altogether 431 breaks, 7.65%), 19p (27 breaks; 6.26%), and 19q (22 breaks; 5.1%). The aberrations involving these chromosomes were of different types, e.g., deletions, translocations, and additions of unknown material. Although simple rearrangements between two chromosomes could be described in some tumors, in the majority of cases it was not possible to identify the partner chromosome in the rearrangement which led us to resort to descriptions such as add(11)(p/q) and/or add(19)(p/q).

**Figure 1:**
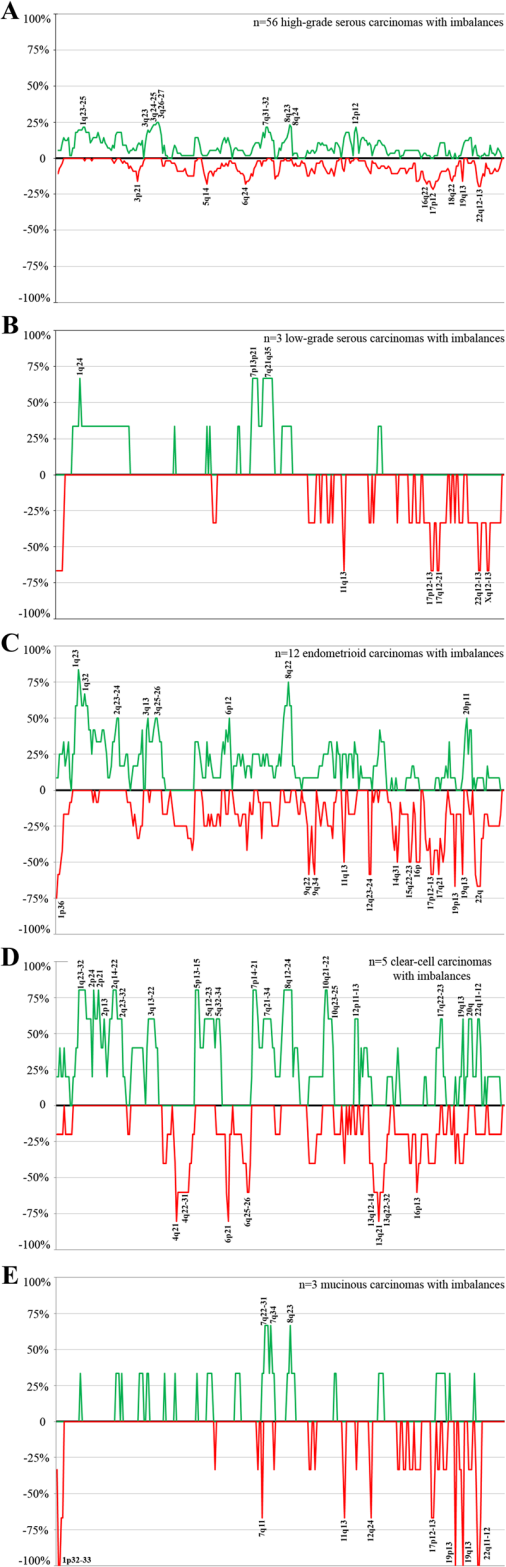
**Profiles of the imbalances detected by HR-CGH in the different subtypes of ovarian carcinomas.** The green color highlights gains, while the red color indicates losses. **A)** high-grade serous carcinomas; **B)** low-grade serous carcinomas; **C)** endometrioid carcinomas; **D)** clear-cell carcinomas; and **E)** mucinous carcinomas.

The HR-CGH analysis of genomic imbalances showed gains and/or losses in 81 tumors (Additional file 1: Table S1), whereas no imbalances were scored in seven samples. No informative results were obtained in another seven tumors due to poor quality of hybridization signals. In the remaining 15 cases, no DNA was available to perform the analysis. Gains were more frequent than losses and amplifications, i.e., more than four-fold gains, were scored in 30 tumors (21 high-grade serous carcinomas, four endometrioid, two clear-cell, one low-grade serous, one mucinous, and one undifferentiated carcinoma). The high-grade serous carcinomas (n = 56 with imbalances) mostly showed gains of 3q26-27 (detected in 25% of the tumors with imbalances), followed by gains of 3q24-25 and 8q23 (in 23%), and 1q31-32, 3q23, 3q28, 7q31-32, 8q24, and 12p12 (all detected in 21% of the tumors; Figure 1). The most frequent losses were scored at 17p12 (21%), 17p11, 17p13, and 22q12-13 (20%), and 5q14, 6q24, and 16q22 (18%). The endometrioid carcinomas (n = 12 with imbalances) showed gains of 1q23 (83% of the endometrioid tumors with imbalances), followed by 1q24 and 8q22 (75%), 1q32 (67%), 1q21-22, 1q25-31, 1q41-42, 8q13-21, and 8q23q24 (58%), and 2q23-24, 3q13, 3q25-26, 6p12, 8q12, and 20p11 (50%; Figure 1). Losses were scored at 1p36 (75%) followed by 19p13 and 22q (67%), and 9q22, 9q34, 12q23-24, 17p12-13, 17q21, 19q13, and 21q22 (58%). The clear-cell carcinomas (n = 5 tumors with imbalances) showed frequent gains from 1q23-32, 2p24, 2p21, 2q14-22, 5p13-15, 7p14-21, 8q12-24, and 10q21-22 (80%) followed by 1q41-44, 2p22-23, 2p13, 2q12-13, 2q23-32, 3q13-24, 5q12-23, 5q32-34, 7p13, 7q21-34, 8q11, 10q11, 10q23-25, 12p11-13, 17q22-23, 19q13, 20q, and 22q11-12 (60%). Losses were most often scored at 4q21, 6p21, and 13q21 (80%) followed by 4q22-31, 6p22, 6q25-26, 13q12-14, 13q22-32, and 16p13 (60%; Figure 1). The mucinous carcinomas (n = 3 with imbalances) showed frequent gains of 7q22-31, 7q34, and 8q23 (67%). Losses were scored at 19p13, 19q13, and 22q11-12 in all three tumors with imbalances (100%) followed by losses of 7q11, 11q13, 12q24, 17p12-13, and 22q13 (67%; Figure 1). The low-grade serous carcinomas (n = 3 with imbalances) had gains mostly of 1q24, 7p13-21, and 7q21-35 (67%), while losses were commonly detected at 11q13, 17p12-13, 17q12-21, 22q12-13, and Xq12-13 (67%; Figure 1). Amplifications most frequently involved chromosomal bands 3q26 (12 tumors) followed by 8q23 (11 tumors), 8q22 (ten tumors), 8q24 (eight tumors), and 3q25 and 8q21 (seven tumors). The high-grade serous carcinomas showed amplifications in 3q26 in ten samples followed by 8q23 (n = 9), 8q22 (n = 8), 8q24 (n = 7), and 3q25 and 8q21 in six samples each. Four endometrioid carcinomas showed amplification, case 84 (Additional file 1: Table S1) in 3q26 and 12p, case 85 in 1q42-44, case 90 in 5p14, and case 93 in 3q25-29 and 6p21-22. Two clear-cell carcinomas showed amplifications: case 97 in 8q and case 100 in 8q13-21 and 8q22-23. The only low-grade serous carcinoma showing amplification (case 75) had it in 1q31, the undifferentiated carcinoma (case 110) showed amplification in 12q13, and a mucinous carcinoma (case 104) had 7q31 amplified.

Both tumors that had received (n = 15) and those that had not received (n = 41) neo-adjuvant therapy prior to the operation showed frequent gains from 3q and 7q; however, the neo-adjuvant group also showed gain of 11q14-22 in 27% of the abnormal cases, whereas the group that did not receive such treatment showed common additional gains from 1q, 2q, 8q, and 12p. Losses were often seen at 8p, 9q, and 10q in the neo-adjuvant subgroup but more commonly at 3p, 5q, 6q, 17p, 17q, and 22q in the normal group.

The average number of copy aberrations (ANCA) index was 39.7 for the high-grade serous carcinomas, 14.7 for the low-grade serous carcinomas, 33.7 for the endometrioid carcinomas, 35.2 for the clear-cell carcinomas, and 20.3 for the mucinous carcinomas. The ANCA index for the tumors (all subtypes taken together) that had received neoadjuvant treatment was 30.85 whereas the value for the group of tumors without neoadjuvant treatment was 39.01.

## Discussion

We report the analysis of 110 ovarian carcinomas of different histological types using G-banding and HR-CGH. We first compared the aberration patterns identified by the two genomic screening techniques using the so-called “classical” pathological classification of ovarian carcinomas advocated by Blaustein [2] where the carcinomas are divided into tumors of types I and II. We also broke down our findings according to the more “modern” classification proposed by Prat [4], which classifies the carcinomas into five different categories.

The overall genomic complexity of the tumors could be assessed both by karyotyping and CGH. The acquired aberrations detected by G-banding resulted in complex karyotypes in the great majority of both type I and type II ovarian carcinomas. Breakpoint clusters were seen first and foremost at 11q, 19p, and 19q. A total of five rearrangements between chromosomes 11 and 19 (in altogether three tumors) were detected in this series; these have been examined in detail previously and reported by us [6]. In our experience with ovarian cancer karyotyping, this is the most common exchange of material between two chromosomes detectable with this technique. Lately, Onkes et al. [13] described a fusion gene corresponding to a der(19)t(11;19)(q13.2;p13.2) in the ovarian cell line SKOV3. The translocation led to a fusion between the Homo sapiens hook homolog 2 (*HOOK2*), mapping on 19p, and the actinin alpha 3 (*ACTN3*), mapping on 11q. We have tested, using PCR-based methods, all 110 samples of the present series for this fusion whose chromosome-level rearrangement could possibly be hidden in the complex and only partially described karyotypes. However, we found no expression of this fusion gene in any of the tumors (data not shown). Possibly other genes mapping to 11q and 19p are involved in fusions; this needs to be further investigated.

With or without recombination with 11q, alterations of chromosome bands 19p13 and 19q13 are among the most frequent cytogenetic changes observed in ovarian carcinomas as first pointed out by Pejovic et al. [14], Jenkins et al. [15], and Thompson et al. [16]. Much later, we subjected a group of ovarian carcinomas showing additional material of unknown origin sitting on 19p/q to examination by microdissection followed by reverse *in situ* hybridization and were able to identify the nature of the rearrangements in more detail [6]. We also performed arrayCGH to identify the genomic regions that were gained or amplified on the structurally rearranged 19p/19q [17]. However, much more remains to be done to understand how the nonrandom 19p/q-changes contribute to ovarian carcinogenesis.

There was no difference in breakpoint clusters among the various histologic subtypes, i.e., they all had tumors with breakpoints clustering to 11q, 19p, and 19q. One should be cognizant of the caveat, however, that the great complexity of the rearrangements, as illustrated by the presence of many markers in most karyotypes, may have hidden rearrangements that could possibly distinguish between the different tumor subtypes. When we looked specifically at the serous subtype, we saw that the low-grade tumors (n = 7) with informative karyotypes (n = 5) were either cytogenetically normal (cases 79 and 80) or had only simple karyotypic aberrations (less than four per karyotype in cases 76, 77, and 78). On the other hand, the high-grade serous carcinomas (n = 73) showed mostly complex and/or incomplete karyotypes if they were not cytogenetically normal, the only exception being case 35 which showed monosomies for chromosomes X, 4, and 10 in three cells. These results are in accordance with the findings in a previous study of ours [12] in which we analyzed borderline ovarian carcinomas of the serous and mucinous subtypes and found only simple karyotypic changes. Furthermore, the results support the suggestion of Prat [4] that low-grade serous carcinomas can be defined as uncommon ovarian carcinomas that show a noninvasive serous borderline component, and that they most likely reflect progression of serous borderline tumors beyond microinvasion.

The most reliable assessment of genomic complexity in terms of gains/losses of chromosomal material was obtained by HR-CGH. We compared tumor subsets according to their ANCA index which was 29.8 for type I and 39.7 for type II carcinomas. The Mann-Whitney U-test showed a significant difference between the two groups (p < 0.05; Table 1). The same test was also performed to see if there were significant differences in genomic complexity among the histologic subtypes (Table 1). The only significant difference was found between high- and low-grade serous carcinomas, with the latter tumors on average showing fewer genomic imbalances (ANCA index 39.7 vs 14.7). Obviously, larger sets of samples for each specific subtype should be tested to confirm or refute this conclusion. The same non-parametric test was also performed to see if there was a difference in the number of acquired chromosomal aberrations between tumors that had received (n = 15) or had not received (n = 41) neo-adjuvant therapy prior to the operation. There was no statistical significance (p = 0.29). However, the Mann-Witney U-test did show a significant difference (p = 0.04) between the number of copy number changes, i.e., the ANCA index, in the two groups.

**Table 1:** Overview of the p values obteined by comparing the ANCA index of the different histological subtypes of ovarian carcinomas, using the Mann-Whitney U-test

	**High-grade serous**	**Low-grade serous**	**Endometrioid**	**Clear cell**	**Mucinous**
High-grade serous	--	p = 0.03372	p = 0.2772	p = 0.624	p = 0.0874
Low-grade serous	p = 0.03372	--	p = 0.1486	p = 0.1771	p = 0.5066
Endometrioid	p = 0.2772	p = 0.1486	--	p = 0.8329	p = 0.3648
Clear cell	p = 0.624	p = 0.1771	p = 0.8329	--	p = 0.2938
Mucinous	p = 0.0874	p = 0.5066	p = 0.3648	p = 0.2938	--

The imbalance patterns detected by HR-CGH were also assessed group-wise with comparisons between type I and type II tumors as well as among all five histologic carcinoma subtypes. The high-grade serous carcinomas (type II) mostly showed gains from 1q, 3q, 7q, 8q, and 12p but losses from 3p, 5q, 6q, 16q, 17p, 18q, 19q, and 22q, whereas the low-grade tumors (i.e., the remainder) showed gains from 1q, 2q, 5q, and 8q whereas losses were seen primarily from 1p, 9q, 11q, 12q, 16p, 17p, 19p, 19q, and 22q. Common imbalances to both type I and type II were gains from 1q, 3q, 7q and 8q and losses from 17p, 19q, and 22q. These results are in agreement with the result of previous imbalance studies of ovarian carcinomas [18-24] as well as with the latest research work performed by the Cancer Genome Atlas Research Network [25] on more than 400 ovarian carcinomas. Furthermore, gain of material from 8q23 and losses from 19q and 22q have been found at high frequencies in bilateral ovarian carcinomas where it was suggested that they probably represent early genomic changes because they were present in tumors of both sides [18]. The very same imbalances were also identified in borderline ovarian carcinomas [12]. The fact that they were present both in “precursor” lesions, i.e., borderline tumors, as well as more advanced stages, i.e., carcinomas, highlights the possibility of an adenoma-carcinoma sequence in ovarian carcinogenesis. The relationship between borderline ovarian tumors and low-grade carcinomas has been investigated at the gene level by many groups [26-28] focusing on the mutually exclusive mutations detected in either the *BRAF* or *KRAS* gene, and a relationship was found between the two groups of tumors. We did not perform any molecular study on our cases; however, our chromosomal results fit the interpretation that a common relationship may exist between the two groups of tumors at the level of both genes and chromosomes.

When looking separately at the imbalance patterns of different subtypes of low-grade tumors, we saw examples of gains and/or losses shared by two or more subtypes but also copy number changes that were only or predominantly present in one subgroup, namely -Xq mostly in low-grade serous carcinomas, +6p, +20p, and -21q mostly in endometrioid carcinomas, and +2p, +5p, +5q, +10q, +12p, +17q, +19q, +20q, -4q, -6p, and -13q mostly in clear-cell carcinomas. These may possibly represent secondary aberrations that influence the tumor differentiation pattern.

## Conclusions

We have shown that aberrations such as gain of material from 8q23 and losses from 19q and 22q, previously described at high frequencies in bilateral and borderline ovarian carcinomas, are also present in tumors of more advanced stages, i.e., carcinomas; this highlights the possibility of an adenoma-carcinoma sequence in ovarian carcinogenesis. The low-grade carcinomas may represent a phenotypically more advanced stage of borderline tumors.

## Competing interests

The authors declare that they have no competing interests.

## Authors’ contributions

FM supervised all the experiments and interpreted the results. LH carried out the cytogenetic and molecular cytogenetic analysis. VMA and BD provided the samples and gave pathologic data. CGT provided clinical information. FM and SH wrote the paper. All authors read and approved the final manuscript.

## Pre-publication history

The pre-publication history for this paper can be accessed here:

http://www.biomedcentral.com/1471-2407/14/315/prepub

## Supplementary Material

Additional file 1: Table S1 Overview of the genomic aberrations found in carcinomas of the ovary, detected by karyotyping and HR-CGH. Click here for file

## References

[B1] Tavassoli FA Devilee P World health organization classification of tumors: Pathology and genetics of tumors of the breast and female genital organs 2003 Lyon: IARC press

[B2] Blaustein A Blaustein’s Pathology of the Female Genital Tract 2002 New York: Springer-Verlag

[B3] Kurman RJ Shih I The origin and pathogenesis of epithelial ovarian cancer: a proposed unifying theory Am J Surg Pathol 2010 34 433 443 10.1097/PAS.0b013e3181cf3d79 20154587 PMC2841791

[B4] Prat J New insights into ovarian cancer pathology Ann Oncol 2012 23 111 117 10.1093/annonc/mdr043 22987944

[B5] Mitelman F Johansson B Mertens F Mitelman database of chromosome aberrations and gene fusions in cancer 2014 http://cgap.nci.nih.gov/Chromosomes/Mitelman

[B6] Micci F Weimer J Haugom L Skotheim RI Grunewald R Abeler VM Silins I Lothe RA Trope CG Arnold N Heim S Reverse painting of microdissected chromosome 19 markers in ovarian carcinoma identifies a complex rearrangement map Gene Chromosome Canc 2009 48 184 193 10.1002/gcc.20628 18973136

[B7] Mandahl N Rooney DE, Czepulkovski BH Methods in solid tumors Human Cytogenetics - a practical approach, vol II, Malignancy and acquired abnormalities 1992 Oxford: IRL Press 155 187

[B8] Shaffer LG Slovak ML Campbell LJ ISCN (2009): An International System for Human Cytogenetic Nomenclature 2009 Basel: Karger S

[B9] Brandal P Bjerkehagen B Heim S Molecular cytogenetic characterization of tenosynovial giant cell tumors Neoplasia 2004 6 578 583 10.1593/neo.04202 15548367 PMC1531662

[B10] Kallioniemi OP Kallioniemi A Piper J Isola J Waldman FM Gray JW Pinkel D Optimizing comparative genomic hybridization for analysis of DNA sequence copy number changes in solid tumors Gene Chromosome Canc 1994 10 231 243 10.1002/gcc.2870100403 7522536

[B11] Micci F Teixeira MR Haugom L Kristensen G Abeler VM Heim S Genomic aberrations in carcinomas of the uterine corpus Gene Chromosome Canc 2004 40 229 246 10.1002/gcc.20038 15139002

[B12] Micci F Haugom L Ahlquist T Andersen HK Abeler VM Davidson B Trope CG Lothe RA Heim S Genomic aberrations in borderline ovarian tumors J Transl Med 2010 8 21 doi:10.1186/1479-5876-8-21 10.1186/1479-5876-8-21 20184781 PMC2838832

[B13] Onkes W Fredrik R Micci F Schonbeck BJ Martin-Subero JI Ullmann R Hilpert F Brautigam K Janssen O Maass N Siebert R Heim S Arnold N Weimer J Breakpoint characterization of the der(19)t(11;19)(q13;p13) in the ovarian cancer cell line SKOV-3 Gene Chromosome Canc 2013 52 512 522 10.1002/gcc.22048 23362175

[B14] Pejovic T Heim S Mandahl N Baldetorp B Elmfors B Floderus UM Furgyik S Helm G Himmelmann A Willen H Chromosome aberrations in 35 primary ovarian carcinomas Gene Chromosome Canc 1992 4 58 68 10.1002/gcc.2870040108 1377010

[B15] Jenkins RB Bartelt D Jr Stalboerger P Persons D Dahl RJ Podratz K Keeney G Hartmann L Cytogenetic studies of epithelial ovarian carcinoma Cancer Genet Cytogenet 1993 71 76 86 10.1016/0165-4608(93)90205-Z 8275457

[B16] Thompson FH Emerson J Alberts D Liu Y Guan XY Burgess A Fox S Taetle R Weinstein R Makar R Clonal chromosome abnormalities in 54 cases of ovarian carcinoma Cancer Genet Cytogenet 1994 73 33 45 10.1016/0165-4608(94)90179-1 8174072

[B17] Micci F Skotheim RI Haugom L Weimer J Eibak AM Abeler VM Trope CG Arnold N Lothe RA Heim S Array-CGH analysis of microdissected chromosome 19 markers in ovarian carcinoma identifies candidate target genes Gene Chromosome Canc 2010 49 1046 1053 10.1002/gcc.20813 20725991

[B18] Micci F Haugom L Ahlquist T Abeler VM Trope CG Lothe RA Heim S Tumor spreading to the contralateral ovary in bilateral ovarian carcinoma is a late event in clonal evolution J Oncol 2010 2010 646340 doi:10.1155 19759843 10.1155/2010/646340 PMC2744120

[B19] Iwabuchi H Sakamoto M Sakunaga H Ma YY Carcangiu ML Pinkel D Yang-Feng TL Gray JW Genetic analysis of benign, low-grade, and high-grade ovarian tumors Cancer Res 1995 55 6172 6180 8521410

[B20] Arnold N Hagele L Walz L Schempp W Pfisterer J Bauknecht T Kiechle M Overrepresentation of 3q and 8q material and loss of 18q material are recurrent findings in advanced human ovarian cancer Gene Chromosome Canc 1996 16 46 54 10.1002/(SICI)1098-2264(199605)16:1<46::AID-GCC7>3.0.CO;2-3 9162197

[B21] Sonoda G Palazzo J Du MS Godwin AK Feder M Yakushiji M Testa JR Comparative genomic hybridization detects frequent overrepresentation of chromosomal material from 3q26, 8q24, and 20q13 in human ovarian carcinomas Gene Chromosome Canc 1997 20 320 328 10.1002/(SICI)1098-2264(199712)20:4<320::AID-GCC2>3.0.CO;2-3 9408747

[B22] Tapper J Sarantaus L Vahteristo P Nevanlinna H Hemmer S Seppala M Knuutila S Butzow R Genetic changes in inherited and sporadic ovarian carcinomas by comparative genomic hybridization: extensive similarity except for a difference at chromosome 2q24-q32 Cancer Res 1998 58 2715 2719 9661879

[B23] Suzuki S Moore DH Ginzinger DG Godfrey TE Barclay J Powell B Pinkel D Zaloudek C Lu K Mills G Berchuck A Gray JW An approach to analysis of large-scale correlations between genome changes and clinical endpoints in ovarian cancer Cancer Res 2000 60 5382 5385 11034075

[B24] Kiechle M Jacobsen A Schwarz-Boeger U Hedderich J Pfisterer J Arnold N Comparative genomic hybridization detects genetic imbalances in primary ovarian carcinomas as correlated with grade of differentiation Cancer 2001 91 534 540 10.1002/1097-0142(20010201)91:3<534::AID-CNCR1031>3.0.CO;2-T 11169935

[B25] Cancer Genome Atlas Research Network Integrated genomic analyses of ovarian carcinoma Nature 2011 474 609 615 10.1038/nature10166 21720365 PMC3163504

[B26] Mayr D Hirschmann A Lohrs U Diebold J KRAS and BRAF mutations in ovarian tumors: a comprehensive study of invasive carcinomas, borderline tumors and extraovarian implants Gynecol Oncol 2006 103 883 887 10.1016/j.ygyno.2006.05.029 16806438

[B27] Ardighieri L Zeppernick F Hannibal CG Vang R Cope L Junge J Kjaer SK Kurman RJ Shih IM Mutational analysis of BRAF and KRAS in ovarian atypical proliferative serous (Borderline) tumors and associated peritoneal implants J Pathol 2013 232 16 22 24307542 10.1002/path.4293 PMC4086471

[B28] Tsang YT Deavers MT Sun CC Kwan SY Kuo E Malpica A Mok SS Gershenson DM Wong KK KRAS (but not BRAF) mutations in ovarian serous borderline tumor are associated with recurrent low-grade serous carcinoma J Pathol 2013 231 449 456 10.1002/path.4252 24549645 PMC4095747

