# Supplementary material for: Genomic profile of ovarian carcinomas
Source: BMC Cancer. 2014 May 5;14:315. doi: 10.1186/1471-2407-14-315 (PMC4101829; doi:10.1186/1471-2407-14-315)
Supplement: Additional file 1: Table S1 — Overview of the genomic aberrations found in carcinomas of the ovary, detected by karyotyping and HR-CGH. [file 1471-2407-14-315-S1.doc]

Additional file: Table S1. Overview of the genomic aberrations found in carcinomas of the ovary, detected by karyotyping and HR-CGH.

| Case num/ lab num | **Diagnosis** | Karyotype | Genomic imbalaces |
| --- | --- | --- | --- |
| 1/59-99 | S; hg | 72~90,XXX,del(1)(q11),der(1)t(1;5) (q43;q11),der(1;16)(q10;p10),i(5)(p10), add(6)(q13),del(6)(q15),del(11)(q12), der(11)t(11;17)(p15;q12),add(12)(p13), i(13)(q10),inc[cp24]/46,XX[4] | rev ish enh(1p35pter,1q31qter,2p13,2p15pter,2q23q37,3q22q28, 4p15,6p12p21,7q31qter,8q23q24,9q21,9q32qter,10p13p15,12q12, 12q13q24,13q12q14,16p12p13,17p11,17q21q25,19p13,19q, 20p11p13,20q11q13,22q11q12),dim(Xq21q22,2q14q22,3p14p24, 4q22qter,5q14q23,6q24qter,7p15p21,8p12p23,9q21q22,10q22q25, 14q22qter,16q,17p12p13,18q21qter) |
| 2/60-99* ^a^ | S; hg | 54~61,add(1)(p21),del(1)(q21),add(4) (p11),add(6)(q27)x2,add(7)(p22)x2, del(12)(p11)x2,+13,der(13;14)(q10;q10), add(15)(p11),der(19)t(7;19)(q11;q13), inc[cp13]/46,XX[3] | rev ish enh(1p31p32,1p36,1q21q24,1q32q43,2p11p21,3q21qter, 5q21q33,6p21p25,7p12p14,7q11q21,7q32q34,8q22qter,11q12q22, 12p12p13,17q22qter),dim(6q23q24, 7p21,13q21q22) |
| 3/00-39^a^ | S; hg | 35~42,del(1)(q21),add(1)(p13),add(6) (q22),inc[cp9]/71~87,idem x2[cp10]/ 46,XX[3] | rev ish enh(1p13p35,1q21q43,2p12p25,2q11q22,2q31,3p21p22, 3p24,3q13q24,3q26q28,4p15pter,5p15,5q13,5q23qter,6p21pter,7, 8p11p12,8q23q24,11p11p14,11q12q24,12p11p12,12p12p13, 12q13q14,12q23q24,13q31qter,15q14q26,16p,16q13qter,17q21qter, 18q12q21,19p13,20p11p12,20q,21q22),dim(Xp11p22,Xq12q25, 2q33q36,3p12p14,4q21q26,4q28q35,6q21qter,8p23,8q21q23, 9q13q34,10p11,10q11q26,14q11q31,17p11) |
| 4/00-55 | S; hg | 39,X,-X,i(3)(q10),-4,add(5)(q13),i(5)(p10), -6,add(7)(p13),add(10)(p11),add(11)(p11), der(13;14)(q10;q10),-17,-21[13]/ 78,idemx2[3]/46,XX[3] | rev ish enh(1,2,3q,5p,7p12p21,7q,8q,9p21p23,9q22qter,10q, 11q12q24,12,13q12q34,14q13qter,15q14qter,17q21qter,18q11q12, 19q13,20,21q22),dim(X,3p,4,5q11q33,6,10p13pter,11p14p15,16,17p,17q11q12, 22q) |
| 5/00-62 ^a^ | S; hg | Culture failure | rev ish enh(Xp,1p13p21,1q,2p21,2q23q32,3q,4q13q21,5p,6q13qter , 7q,10p11p14,10q21q22,11p14,11q13q24,12,13q13q22,20q11q12, 21q21q22),dim(Xq21q26,1p34p36,2p25,3p21p26,4p15p16,5q12q14, 5q32qter,6p21p24,7p21pter,8p,8q11q12,9q21qter,10q23qter,11p15, 11q12q13,14q11,14q13qter,15q,16p11pter,17p12pter,18p11,18q, 19p13,19q13,22q12q13),amp(5p15,10q22) |
| 6/00-94 ^a^ | S; hg | 56~81<3n>,XXX,der(1;16)(q10;p10)x2, dup(1)(q21q32),+dup(1)(q21q32),del(2) (p16),t(2;6)(q33;q13)x2,-4,-4,-5,-5, -7, -7, -8,-9,-9,-9,add(11)(p14)x3,dup(12) (q13q24)x2,+13,add(13)(q34)x2,-14,-14, -15,der(15;22)(q10;q10),+16,+17,der(17) t(5;17) (q11;p11)x2,-18,+5~11mar[8]/ 46,XX[10] | rev ish enh(2q32q34,3q12q13,3q25q27,6p12p21,6q12,6q13q21, 6q22q23,7q31q32,8q22qter,11q14q22),dim(7q11,8p12pter,9q22, 10q23q24,10q26,12q23qter,15q15q21,15q22q24,16p12p13, 16q22,17p,17q11q22,18q21q22,19q13,22q) |
| 7/00-129 | S; hg | 87~141,dup(1)(q21q31),add(3)(q25), add(4)(q31),add(6)(q21),inc[3]/ 46,XX[13] | rev ish enh(Xq,1q21q31,2p23p24,2q22q32,3p21pter,3q13qter, 5p13pter,6p23p24,7q21q34,8q21qter,10q22q23,11q13q23, 12p12pter),dim(1p35p36,3p12p21,4q31q34,4q35,6q,7p14pter, 8p12pter,11p14,12q21qter,13q14qter,16q13qter,17p11p12, 17q11q21,18,19q13,20q11q13,21q,22q11q13),amp(8q22qter) |
| 8/00-180 | S; hg | Culture failure | No DNA available |
| 9/00-206 | S; hg | 116~162,add(4)(p15),add(5)(p13), del(7)(q31),der(8)t(8;13)(p23;q14), i(9)(q10),add(11)(p15)x2, add(15)(p11)x2, inc[6]/46,XX[94] | rev ish enh(Xq21,1p13p34,1q21q32,2p,2q11q12,3p12p14,3q, 5p13pter,6p23,7q21,7q31q32,8p11p12,8q,9q21q33,11p11p14,12p11p12,12q12q21,14q24qter,20q12qter,21q),dim(Xp11pter,2q35q37, 5q11q15,5q23q32,5q34qter,6q24q26,8p21,10q11q22,11p15, 11q12q13,15q11q25,16,17,19p13,19q13,22q) |
| 10/00-289^a^ | S | 46,XX[22]/92,XXXX[5] | No DNA available |
| 11/00-522 | S; hg | 46~49,add(11)(q22),add(14)(p11), add(16)(q23),add(19)(q13),inc[6]/ 89~94,idemx2[2] | rev ish enh(1q21q32,2q14q32,3q23q27,8q23q24,10q25,12p11p13, 12q14q21,13q21q33,14q11,20p11p13,20q11q13),dim(Xp11, Xq26q27,3p14,3p21,5q13q14,8p22p23,16q) |
| 12/00-672 | S; hg | 59~62,inc[5] | rev ish enh(Xp11,Xq25q26,1p13p31,1q25q43,2p16p22,2q14q35, 3p12p14,3q,4q12q21,6p11p12,6p21p24,7p11p12,7q11q36,8q11q23, 10p11p14,11q13q24,12p11p13,12q12q24,13q13q34,14q11q32, 17q11q12,17q21qter,18p,19q13,20,21q21),dim(Xp22,1p33pter, 3p21p25,4p15pter,4q22qter,5p14pter,5q11q14,5q23qter,6q24qter, 7p14p21,7p21pter,9p12p23,9q,10q11q24,11p12pter,15q11q21, 16p11pter,17p11pter,18q12,18q22,22q12qter) |
| 13/00-732* | S; hg | 78~88<4n>,XXX,-X,add(1)(p36),-2, add(4)(p15),del(6)(q14)x2,add(7) (p21)x2, der(7)add(7)(p15)add(7)(q36),-10,-10, del(10)(p12)x2,add(11)(p14)x2,-13,-14, -15,-17,-17,-18,der(19)t(19;21) (q13;q11)x2,inc[10]/46,XX[6] | rev ish enh(Xq21,Xq26qter,1p13p32,1q21q31,1q32q42,1q43,1q44, 2p11,2p13,2p21pter,2q11q14,2q22q32,2q35q36,3p12p21,3p23pter, 3q12q24,3q25qter,5p13pter,5q15qter,6p,7p13p15,7q21q34,8q, 9p21p24,9q13q21,10p11,10q11q21,10q22,10q24qter,11p11, 11p12p15,11q13qter,12p12pter,15q12q26,20p11p13,21q21q22), dim(Xp11pter,2q21,2q32q33,2q37,4q,5q12q14,6q14qter,7p21pter, 7q35qter,10p12pter,11p15,12q,13q,14q21qter,16p,16q13qter,17,18, 19p,19q13, 22q11q13),amp(8q21qter) |
| 14/00-757 | S; hg | 63~69,X,+add(1)(q21),+2,+add(2)(q12), del(2)(q33),+7,+del(11)(q14),+12, +add(12)(p12),add(13)(p11),i(14)(q10), inc[27] | rev ish enh(1q23q43,2p,2q22q31,3p12,3p14p21,3q12q29,4p15, 5p12pter,6p,7p11p22,7q11q22,7q31,8p12p21,8q11q24,10p,10q11, 11p12p15,12p,12q12q23,13q,19q13,20),dim(X,1p32pter,3p21p26, 4q28qter,5q12q21,5q21qter,6q14,6q15qter,9p,9q,10q26,11q12q13, 11q23,14q,15q11q21,15q21q24,16p,16q13qter,17p,17q11q23, 17q23q25,18p11,18q,19p13,19q13,21q22,22q),amp(12p11pter) |
| 15/00-764 | S; hg | 54~72<3n>,X,add(X)(q28),add(4)(p11), del(7)(p14),der(7)t(7;7)(p15;q11), del(12)(q24),del(16)(q23),add(19)(q11), add(19)(q13)x2,inc[2]/46,XX[39] | rev ish enh(Xq21q23,1p13p32,1q,2p16p21,2p22pter,2q22q32, 2q34q35,3p12p21,3p22pter,3q,4p13p16,4q12q21,4q31q35,5p12pter, 5q33q34,6p11p21,6p22pter,6q12q14,7p11p21,7q, 8p11p12,8q,10, 11p11p13,11q12q23,12p,12q12q13),dim(Xp22,1p35pter,2q37,3p21, 5q13q31,6q15qter,8p21p23,9p12p21,9p23pter,9q13q21,9q33qter, 11p14pter,11q23qter,12q22q24,13q14q21,13q21q32,13q34,14q21, 15q15q21,16p11pter,16q,17,18q21qter,19p,19q13,22q), amp(8q21qter,11q14q22) |
| 16/00-801 | S; hg | 63~106,add(4)(q34),add(6)(q12), add(14)(p11),inc[3]/46,XX[10] | rev ish enh(1p22p31,1q,2p12p14,2q22q24,2q32q33,3p12p14, 3q24q28,4q12q24,5p12p14,5q11q12,6q12q21,6q22,7q31qter, 8q13qter,10p11p14,10q11q21,11p13p14,11q14q22,12p11p12, 13q21q22,16p11p13,16q12q21,17q22qter,18q12,20q13), dim(Xp11p22,Xq12q23,Xq25q27,3p21,5q14q23,6p23pter,7p, 7q11q31,8p12p21,8p22p23,9q21q31,10q21qter,11p15,11q13, 12q21qter,13q12q13,13q33q34,14q11q13,14q21qter,15q22q26,17p, 17q11q21,18p11,18q21qter,19p13,19q13,22q) |
| 17/00-914 | S; hg | 46,XX[6] | rev ish enh(Xq21,Xq26q27,1p13,3p12,3q12q13,4p15,4q13, 4q22q26,4q27q28,5q14q22,13q21q31),dim(1p32pter,1q32,2q37, 6p21,7p22,7q11,9q34,11q12q13,12q24,16p11pter,16q21qter,17p, 17q11q21,17q22qter,19,20q11q13,21q22,22q11qter) |
| 18/00-1012* | S; hg | 39~41,X,del(X)(q22),add(1)(p36),der(1) add(1)(p34)del(1)(q31),add(2)(q37), add(3)(q29),del(3)(q21),-4,-5,add(6) (q15)x2,der(9)(9pter→9q34::11q13→ 11q25::11q23→11q25),-10, i(13)(q10),-15, -16,-17,-17,der(19)t(16;19)(q12;q13), i(19)(q10),-21,-22,+4~8mar[7]/ 72~82,idemx2[3] | rev ish enh(Xp11p22,Xq12q21,1p13p33,1q21q43,2,3p12p14,3q, 4p13pter,5p13pter,5q31q34,6p12p23,7p12p13,7q11q36,8q11q24, 9p13p23,9q21q34,10q11q21,11p11p14,11q12q22,12p,12q12q13, 12q14,12q21q24,13q21q34,14q11q21,14q21q24,15q25q26,18p11, 18q11q21,20p,20q12q13),dim(Xq24qter,3p21p26,4q13q35,5q12q23,6q16qter,7p15pter,8p22pter,10p13pter,10q22q25,11p15,11q23qter, 13q12q21,14q32,15q11q22,16q12q22,17p11p12,17q11q12, 18q22q23,21q,22q),amp(3q26qter,18q12,20p12p13) |
| 19/00-1030 | S; hg | Culture failure | No DNA available |
| 20/00-1046 | S; hg | Culture failure | rev ish enh(Xp11,Xp21p22,1p21p31,1q22q32,2q12q32,3p12p23, 3q,5p12p14,5q21qter,6p,6q12q22,8p11p21,8q11q22,8q23qter, 9q21q32,10p14p15,10q21,10q22q23,11p11p14,11q12q23,12p11p13,12q12q13,12q14q21,13q22qter,20),dim(1p34pter,1q41,1q42q43, 1q44,2p23pter,2q35qter,4q13q28,5q11q14,6q24qter,7p14p21,7p22, 7q11q21,10q25qter,11p14p15,11q23qter,12q24,13q14q21,14q21q24, 15q13q15,15q23q26,16q21qter,17p,17q11q24,18q12qter,19p13,19q,22q13),amp(6p22pter,8p11,8q11,11q14q22) |
| 21/00-1091 | S; hg | 42~47,add(16)(q12),add(17)(q22), add(19)(q13),inc[13] | rev ish enh(10p15, 10q11q22,17q21q25),dim(10q25q26) |
| 22/00-1137 | S+E; hg | 49~64,add(1)(p36),der(1)del(1)(p32) add(1)(q25),dup(1)(q25q42),del(3)(p11), add(5)(p15),add(10)(p11)x2,inc[cp5]/ 46,XX[91] | rev ish enh(Xp21pter,Xq21,1p13p22,1p32,1q23q41,2p13,2p14p16, 2p23p24,3p12p21,3p23p26,3q,4p14p16,5p,6p11p24,6q12,7q21, 7q22q31,8p11,8q,9p13pter,9q21,10p11pter,10q21,11q13q23,12p12, 12q14q21,14q11q21,14q24q31,15q24q26,18q22q23,20p11p13, 20q13,21q21),dim(Xq23qter,2q,4q,5q12q23,5q32qter,6q16q23, 7p15pter,8p12p23,10q22q23,10q26,11p14p15,13q12q13,13q21q34, 15q11q21,16p11p13,16q12q21,17p11p13,17q,18q11q21,19p13, 19q13,22q),amp(1q31,3p12,3q12q13,3q21q26,5p13p15,8q13q21, 8q22q23,9p23) |
| 23/01-77 ^a^ | S; hg | 74~83,i(5)(p10),del(6)(q21),add(11) (p14),add(17)(p12),der(19)t(1;19) (p13;p13), inc[9]/46,XX[10] | rev ish enh(Xq27,1p13p32,1q21q32,2p,2q11q24,3p12p25,3q12q26, 4p15pter,5p14p15,6p12p21,6p24,6q12q15,7p13p21,7q31qter,8q, 11p14p15,11q13q14,11q24,12p12pter,12q13q21,13q21q33, 14q11q12,15q24q26,16p11p13,17p12,18q11q21,20p12,21q21),dim (Xp, Xq12q25,1p34pter,1q41qter,2q31qter,4p13p15,5q11q22,5q23, 5q31,6q16qter,7q21,8p,9p,9q22q32,10p12p13,10q26,11q22q23, 12q23qter,13q12q21,14q21q31,15q13q21,16q21q22,17q,18q22qter, 19p13,19q13,20q11q12, 22q12q13),amp(7q32q36,8q21qter) |
| 24/01-163 | S; hg | 46,XX[4] | rev ish enh(Xq21q27,1p13p31,1q22qter,2p,2q11q24,3q13qter, 4q13,5p,5q11q13,5q21q23,5q31q34,6p12p21,6p22p23,6p24, 6q12q21,7q21,7q22q32,7q33q34,8p12p21,8q11q21,8q21qter, 9p13p23,9q21q22,10p11,10p13,10q11q21,10q22q25,11p11p14, 11q13q23,12q12,14q12q13,20p11p12),dim(Xp11,1p34pter,3p12, 3p14p21,4q21q26,6q22q27,7p11p22,7q11,8p23,9q33qter,12p12p13,12q21q23,13q12q14,14q21q31,15q11q21,16,17p,17q11q21,18,19q, 21q21q22, 22q12q13),amp(3q25q26,8q22q24) |
| 25/01-169 ^a^ | S; hg | 46,XX[48] | rev ish enh(2q22q24,2q32,5p14,5q14,5q21,6q15q16,8q21,8q22q23, 11q22,13q21q31,21q21),dim(Xp11,1p32p36,7q11,7q35q36,9q22, 12q24,14q31,15q23q24,17p12p13,17q11q21,19p13,19q13,20q11q12,22q11q13) |
| 26/01-295 | S; hg | 42~45,XX,t(2;11)(q31;q14),+7,+14,-20, add(20)(q13),+mar[7]/83~85,XXXX, idemx2[4]/46,XX[1] | rev ish enh(2p23p24,2q14,2q22q23,3p23p24,3p25,5p14,6q15q16, 8p21,8q13,8q21,8q22q24,9p23,11p14p15,11q22,13q22,20q), dim(1p32p36,10,12p12,12q24) |
| 27/01-431 | S; hg | 44~48,X,del(X)(q23),-1,-1,add(2)(p21), der(3)(3qter→3p11::?::1p22→1pter), add(8)(p22),-9,del(11)(q22),der(12) t(1;12)(p13;p11)dup(1)(p13p36),+der(12) (12qter→12p11::?::Xq28→Xpter), add(16)(p13),add(17)(p11), add(19)(p13)[4]/ 85~95,idemx2[3] | No DNA available |
| 28/01-548 | S; hg | 55~80,inc[8]/46,XX[11] | rev ish enh(Xq21,Xq23q24,1p12p32,1p36,1q,2p,2q11q33,3p12p14, 3q,4p,4q12q26,5p14,5q14q22,6p11p21,6p22p25,7p,7q11q35, 8q13q21,8q22q24,10p11,10p13pter,10q11q23,10q24,11p12p15, 11q22,11q23qter,12p,12q12q22,18p,18q11q12,20p11p13,21q21),dim(Xp,2q37,3p21pter,4q35,5q23q32,5q34qter,6q16q27,7q11,8p,9p, 9q21qter,10q26,11q12q13,12q24,13q,14q12qter,15q11q24,16p, 16q22q23,17,18q21qter,19p13,19q13,21q22,22q) |
| 29/01-578* ^a^ | S; hg | 105~110<5n>,XXX,-X,-X,+1,-2,+add(5) (p15),+del(6)(q11),der(6;19)(p10;q10)x2, +i(7)(q10),-8,-8,-9,-10,-11,+12,add(12) (q12),-13,add(13)(p11),-14, -14,add(14) (p10),-15,-15,add(15)(p10),der(17)t(6;17) (p11;p13)x2,+i(18)(q10),der(19)t(6;19) (p12;q13),der(19)t(19;22)(q13;q11)del(19) (p13),-21-22,-22, +4~10mar[cp14] | rev ish enh(1p12p31,1q21q31,1q32q41,2p11,2q11,3p12p13, 3p22p23,3q12q13,3q21q26,4p14p15,4q12q13,4q26q27,5p12p15, 5q11,5q12,5q14q23,5q33,6p,6q12,9p23pter,9q13q21,17p11,18, 19p13,19q13,20p11,20q11,21q21),dim(6q25q26,9q34,14q22q24, 15q14q21,15q22q24,16p11pter,16q21qter,22q11q13) |
| 30/01-1000 | S; hg | Culture failure | rev ish enh(1p12p21,1p31p34,1p35p36,1q21q31,1q32q43,2p11p24, 2q11,2q21qter,3q24q26,5p13,6p11p25,6q12q13,6q16q23,7p11p21, 7q11,8p11p12,8q,9q31q32,10p11p14,10q11,10q22,10q24qter, 11q13q23,12p13,12q21q24,13q21qter,14q21q23,16q12q13,19q, 20p11p13,20q,21q21q22,22q11q13),dim(Xp,Xq23q26,3p14p26, 3q13q21,4p13p15,4q28q32,5p14pter,5q11q13,7p21p22,7q35q36, 8p22p23,11p15,11q23q25,13q12q21,15q11q21,15q23q26,16q21qter, 18q12q21,18q22q23),amp(2q36q37,8q23q24, 20q13) |
| 31/01-1020 | S; hg | 46,XX[3] | rev ish enh(1p13p21,1p31pter,1q21q24,1q41,2p13p22,2q11q22, 2q36q37,3p13p14,3p24p25,3q13q23,3q24qter,5q21q23,5q31qter,6p,7p11,7q11q22,7q33qter,8p11,8q,9q,10p13pter,10q23qter,11p, 11q12q23,12q24,14q24qter,16p12,17q22qter,18q,19q13,20q, 21q11q21),dim(X,1q25q32,2p23p25,3p21p22,4q22q28,4q32q35, 5q11q14,6q12q14,6q25qter,7q31q32,9p13p24,11q23q25,12q12q21, 13q21qter,14q13q22,15q11q26,16q21qter,17p11p13,17q11q21, 18p11,21q22,22q),amp(7q36,8q21qter) |
| 32/02-19 ^a^ | S; hg | 63~68,XX,-X,-1,-2,add(3)(p12),-4,+6, add(6)(q27),-7,add(7)(p21),-8,-9, add(11) (p14),-13,-13,-14,-15,add(15)(p11), add(16)(q22),-19,+9~15mar[cp13]/ 46,XX[8] | rev ish enh(2p11p12,2q14q34,3q12q13,3q21q27,6q12q21,6q22q23, 7q11qter,8q11q21,8q23qter,9p,12q14q15,12q21,14q21,18q12),dim (1p31p36,1q41q43,3p13p24,4q33qter,5q34q35,7p,8p12p23,9q22qter,10p12p13,10q22q24,10q26,11p15,11q12q13,11q23,12q24,13q12q31,15q13q21,15q22q24,16p,16q13q24,17,19p13, 19q13,21q22,22q) |
| 33/02-79 | S; hg | 46,XX[3] | rev ish enh(Xp21,1p13p31,1q23q31,2p16,2p21p22,2p22p25, 2q24q34,3p12p14,3p21p26,3q,4p12p15,4q13q28,5p14p15,5q32q33, 6q12q24,8p11p21,8q,9p13p23,9q13q21,10q21,10q25qter,11q13q24,12q14q21,13q21q33,15q24q26,16q12q13,17q24q25,20q13,21q21), dim(Xq27,Xq28,1p33p36,1q32,2p11p12,2q12q22,2q37,4q31qter, 5q11q31,7p13pter,7q11,7q22qter,8p22p23,9q34,10p12,10q23q24, 11p14p15, 12q24,14q22q31,15q21q23,16p,16q23q24,17p11p13, 17q11q22,18p11,18q11q21,19,20p,20q11q12,21q22,22q11, 22q12q13),amp(2q32,3q24q28,8q12q23) |
| 34/02-93 | S; hg | 46,XX[93] | rev ish enh(Xq21,1p13p31,1q31,2p23p24,2q21q24,2q31q32, 3p12p14,3p21p23,3p25,3q12q13,3q24q26,4p12p15,4q12q22,5p, 5q14q23,6q12q22,7p13p21,7q21q31,8p11p12,8q,9p13p24,10q21, 11p11p15,11q14q22,12q13q21,13q21q32,18q12q21,20p12, 21q11q21),dim(Xp11,Xq25q26,1p32pter,2q37,4q24qter,5q11q14, 5q23q31,5q35,7q11,7q31qter,8p21pter,9q,10p12,10q22qter, 11q12q13,12p11p13,12q22q24,13q12,14q12q24,14q31q32, 16p11p13,16q13q24,17,18p11,19p13,19q13,20q12,22q11q13), amp(5p) |
| 35/02-164 ^a^ | S; hg | 41~43,X,-X,-4,-10[3]/46,XX[62] | rev ish enh(1p35pter,1q21q24,2p16,2q14q21,3p22p23,3q13q26, 3q26q27,8q23q24,11q13,15q23q25,20q12q13),dim(4q12q13, 4q25q27,4q28,6q21q24,6q25,18p11,18q22, 18q23) |
| 36/02-233* | S; hg | 62~71,XX,del(1)(p32),add(5)(p13), add(12)(p11),der(19)t(11;19)(q13;p13), i(19)(p10)inc[cp8]/46,XX[2] | rev ish enh(1p32p33,1q21q31,2p13,2p22pter,2q11q24,3p12p21, 3p24,3q12q24,3q26qter,4p15p16,5p,6p12p21,6q12q21,7q31, 7q35q36,8q13qter,9p,9q13q21,11q13qter,12p11p13,19p13,19q13, 20p11p13,20q),dim(Xp11pter,Xq13qter,1p21,4q22q26,6q24qter, 8p12pter,9q22q31,10p11p13,10p14p15,10q11q24,10q25qter, 12q22q24,13q, 14q22q24,14q31q32,15q11q23,16q12q23,17p11pter, 17q12q21,21q21q22) |
| 37/02-248 | S; hg | 59~63,XXX,add(2)(q36),add(3)(q28), add(5)(p15),add(19)(p13),der(19)add(19) (p13)add(19)(q13),inc[2]/46,XX[5] | rev ish enh(1p13p32,1q21q31,2q13q37,3p12p21,3q13qter,4q13q21, 4q27q31,5p13p15,5q33q34,6p22p23,8q12qter,9q13q21,9q32q33, 11p12p14,11q13q23,12q14q23,13q22q31,13q32,14q13q22,20q13), dim(Xp,Xq12q25,1p36,2p14p22,2p24p25,4q31qter,5q12q14, 6q21q22,6q23q26,7p,7q11,8p12pter,10p11pter,10q23q24,12p11pter,12q12q13,15q,16p, 16q21qter,17p,17q11q21,18q21qter,19p13,22q), amp(3q25q26) |
| 38/02-333 | S; hg | 47~49,XX,+8,+9[2]/49,idem,+5,-6,+7[4]/ 54,idem,+3,+5,+6,+7,+14,+17,+19[5] | rev ish enh(1p36,2q37,3p14p21,3p24,3q13q23,3q27q28,4p16,5,7,8, 9p12p23,9q,11q13,12q24,14q31q32,16p12p13,16q22q23,17p11p13, 17q11q21,17q23qter,19,22q11q13),dim(6,21q21) |
| 39/02-363* | S; hg | 65~68,XX,-X,+1,+2,del(3)(p13),+4,add(4) (p12)x2,add(5)(p15),-6,+7,add(7)(p15),-8, -9,-12,-13,-14,-15,-17,-18,der(19)add(19) (p13)add(19)(q13),+20,+20,-21,-22, +2mar[9] | rev ish enh(Xq21qter,1p12p32,1q,2,3p12p14,3q,5p,5q14q15, 5q21q22,7p13pter,7q21q36,8p11p12,8p23,8q,9p21pter,10p11p12, 10p13pter,10q,11p14p15,12p,12q12q13,13q22qter,19p13,19q13,20),dim(Xp,Xq12,Xq13,3p21,4p13pter,6,9q21qter,11q12q13,12q23q24, 13q12q21,14q,15q,16p11p13,17,18,19q13,21q,22q) |
| 40/02-466 | S; hg | 73~81,del(1)(p22),add(1)(q12),add(4) (p11),del(6)(q15),i(8)(q10),add(11)(q22), inc[6] | rev ish enh(Xp11,Xp21,Xq12q13,Xq21qter,1p12p21,1q21q32, 2p21pter,2q21q36,3p12p21,3q12q27,4q12q23,5p,5q15q33,6p,6q12, 7q31q36,8p11p12,8q,9p21pter,10p,10q11q21,11q13qter,12p11pter, 14q12,15q24qter,18q11q21,20p11p13,20q13),dim(1p31p32, 1p34pter,2q13q14,4p16,6q23qter,7p14pter,8p21pter,9p12p13,9q, 10q22q25,11p15,12q12q24,13q12q22,13q31qter,14q21q24,14q32, 15q13q15,16p11p12,17,18p11,18q22q23,19p13,19q,22q) |
| 41/02-478 | S; hg | Culture failure | No DNA available |
| 42/02-489* | S; hg | 57~61<3n>,XX,-X,-1,-1,i(2)(q10),-8,-9, add(9)(p13),-15,add(15)(p11),add(16) (q23),der(19)(19pter→19q11::19q11→ 19p13::10q25→10qter),+r,+3~11mar, inc[9]/115~122,idem[2] | Not informative |
| 43/02-495 ^a^ | S; hg | Culture failure | rev ish enh(1q,2p11p12,2p23p24,2q14,2q21q32,2q35q36,3p12p14, 3p22p26,3q12q22,3q26q27,5p14p15,5q11q23,5q32q33,6q14q23, 8p21p23,8q13q24,10p11,10p13p15,10q11q21,10q22,10q24q25, 12q21,13q12,13q21q31,18q12q22),dim(Xp,Xq12q13,Xq21qter, 1p13pter,4p12p15,4p16,4q12,4q13qter,9p12p23,9q21qter,11,15,16, 21q21qter,22q) |
| 44/02-541 | S; hg | 86~91,XXXXdel(1)(p13),inv(3) (p14q29)x2,-7,-10,-22,-22[10] | rev ish enh(1q23q24,5p13,5p14,5p15,5q11,5q21q23,8q21,8q22q23, 8q24,10q25q26,20p12),dim(1p31p35,21q21,22q11q13) |
| 45/02-735 ^a^ | S; hg | Culture failure | Not informative |
| 46/02-781* ^a^ | S; hg | 67~78,XXX,der(2)del(2)(p21)add(2) (q37),del(7)(q22),del(7)(q32),add(8) (p11),add(12)(p12),der(19)t(X;19) (q13;q11), inc[cp8] | rev ish enh(Xq12q13,Xq22q27,1p,1q21q31,2p,2q11q14,2q34q36, 3q13q22,3q24q26,4p15p16,5p13pter,5q31qter,6p22p24,6q24, 6q25q26,7p11p22,7q11q22,7q33q36,8q21qter,11p11p14,11q12q23, 12p11pter,14q11q13,15q11q21,17q23q25,19p13,20p11p13,20q), dim(3p13,3q28qter,4q26q28,4q33qter,5q13q23,8p12p22,8p23, 9p21p23,12q13q22,12q24,13q14q31,14q24,16q21q23,17p,17q11, 18q12q23,19q13) |
| 47/02-814* | S; hg | 71~79<3n>,X,-X,-X,+1,add(1)(p31), der(1;6)(p10;p10),der(1;16)(p10;q10), der(2)t(2;11)(q36;q22),+del(3)(p21),add(4) (q35),del(6)(q21),der(7)?del(7)(p21)add(7) (q36),i(8)(q10),-9,add(11)(q14)x2,i(14) (q10), der(14;15)(q10;q10),add(16) (p13)x2,add(19)(q11),der(19)(11qter→ 11q14::19p13→19q13::11q14::19p13→ 19q13::19p13→19q13::11q14→11qter), der(19)t(11;19)(q14;p13)del(19)(q13), add(21)(p11),+7~15mar[cp11]/46,XX[1] | rev ish enh(1p13,1p21p22,1p31,1q23q31,2p15pter,2q22q34,3q12q13, 3q22q24,3q24q25,4q12q22,8q,10p,10q24q26,11q14q22,19q13, 20p11p13,20q12q13),dim(Xp,Xq13q21,4p16,4q31qter,5q11q14, 5q23qter,9q22q32,9q33qter,11p12p14,13q14,14q13q21,16q21q23, 16q24,17,19p13,22q13) |
| 48/02-1088 ^a^ | S; hg | 46,XX[24] | no imbalances |
| 49/02-1141 | S; hg | 48~54,der(1)del(1)(p34)del(1)(q31), add(3)(q24),inc[11]/46,XX[5] | No DNA available |
| 50/02-1144 ^a^ | S; hg | 38~44,add(16)(q24),add(19)(p13), add(19)(q13),inc[10]/82~85,idem[2]/ 46,XX[95] | rev ish enh(1p22p31,1q23q31,2p15p16,2p23pter,2q31q36,3p12p14, 3q21qter,6p12,6q23q24,8q12qter),dim(Xp11p21,Xq25,1p32p35, 4q24q25,4q33q35,7p22,7q11,8p12p23,10p11p13,11p15,11q13, 12q23,16p12pter,16q21qter,17p11,17p12p13,17q12q21,18q11, 18q12q22,18q23,19p13,19q,20q11q13,21q21q22,22q13) |
| 51/03-5 | S; hg | 63~79,inc[3]/46,XX[5] | rev ish enh(2q22q32,3q,7p13p14,7q21q31,7q31q34,8p11,8q,9p, 9q13q22,9q22q34,12,17p13,20q11,20q12q13),dim(Xp,Xq25,Xq27, Xq28,1p33p34,3p14p25,4p,4q12q13,4q13q23,4q24q31,4q31q32, 5q11q21,7p21pter,11p11pter,11q12q14,11q22q23,13q12q22, 13q31qter,15q,16p11,17p11p12,17q,19, 21q11,21q21qter,22q) |
| 52/03-37 ^a^ | S; hg | Culture failure | No DNA available |
| 53/03-274 ^a^ | S; hg | 75~80,inc[6] | No DNA available |
| 54/03-420 | S; hg | 34~40,del(1)(p32)x2,-2,-2,-3,del(3)(p21), -5,-5,del(6)(q23),+del(7)(q31),add(7)(p21), -8,-8,-9,-9,-10,add(11)(q22), inc[27]/ 46,XX[8] | No DNA available |
| 55/03-751* | S; hg | 62~79,del(11)(q23),der(19)t(15;19) (q21;p13),+r, inc[17]/46,XX[1] | rev ish enh(1p31p33,1q21q31,1q32q43, 2q22,2q31q37,3p12, 3q12q27,5p13p15,5q21qter,6p11p12,6q12,6q13q22,7q32q36,8q, 9p13p24,10p11p13,10q11q21,11p11p14,11q12,11q13qter,12p11p13,12q12q15,13q14q22,14q11q22,14q24q32,16p12pter,16q12q21, 18p11,20q11qter,22q11q12),dim(Xp11p22,1p36,2p16pter,3p22p26, 4p12p16,4q12,4q34qter,5q11q15,6p22pter,6q22qter,7p11,7p14p22, 7q11,7q21,8p11pter,9q22qter,10p14p15,10q22q24,11p14p15, 12q21q24,13q31qter,15q,17p,17q11q21,17q22qter,18q21qter,19p13),amp(3q13,3q24q26,11q22q23,11q23qter,14q12q21) |
| 56/03-819* ^a^ | S; hg | 109~120<5n>,+del(1)(p21),der(1)add(1) (p36)del(1)(q31)x2,i(1)(q10),+del(5)(q14), -6,add(6)(q13),add(10)(p15),der(11) add(11)(p13)del(11)(q21),-12, i(12)(q10), -13,-14,-14, -15,-16,-16, -17,-17,dup(17) (q21q25),der(19)(20p13→20q13::19p13→19q13::8q22→8qter),-19,-20,+21,+i(21) (q10)x2,+r,+9~24mar[cp14] | rev ish enh(1p13p31,1q21q31,2p11,2p12pter,2q11q36,3p12p14, 3p22p24,3q,5p14p15,5q14q23,6q16,7p13p22,8q22qter,9p13pter, 11p14p15,11q22,12p11p13,12q15,12q21q22,13q21q33,17q22q25, 21q21,21q22),dim(X,9q22q33,9q34,14q,15q,16q13q24,17p, 17q11q21,18p11,19p13,19q13,22q) |
| 57/03-938 | S; hg | 93~111,i(17)(q10),+1~3r,+3mar, inc[cp14]/46,XX[1] | rev ish enh(Xq21,Xq23,1q23qter,2p13,2q22q36,3p12p14,3p22p24, 3q,4p,4q12q21,5p,5q11,5q14q35,6p12p21,6q12q13,7q21,7q31,8p,8q11q24,10q21,11q14q23,12p,12q12q23,14q21,18p11,18q11q12, 19q13,20),dim(Xp11p21,Xp22,1p31pter,6q21q22,6q23qter, 7p11p21,7p21pter,7q11,9p,9q21q33,9q34,10p12p14,10q26, 11p12p14,11p15,11q12q13,13q12q13,14q32,15q11q24,16q,17p, 17q11q21,17q23q24,17q25,18q21qter,19p13,19q13,21q22,22q), amp(5p14p15,20p12pter) |
| 58/03-966 | S; hg | 69~74,inc[15] | rev ish enh(Xq27qter,1p13p31,1q21q22,1q31qter,2p12p13,2p21p22, 2q22q33,3p12p14,3q,5p14p15,5q33q34,7q31,7q32qter,8q,9p,10p11,10q11q22,10q24qter,11p12p13,14q13q22),dim(Xp11pter,1p33pter, 2q35qter,4q13q26,4q31qter,5q12q14,6q22q25,7p11p13,7q11, 8p22pter,9q33qter,10p15,11p15,11q23qter,12q22qter,13q12q21, 14q31q32,15q14q24,16p12,17p,17q11q22,18q21q22,18q22qter,19q, 20p12p13,20q12q13,21q21qter,22q12qter),amp(3q26qter) |
| 59/03-972* ^a^ | S; hg | 69~75,?del(X)(q22),+3,del(3)(q13), add(3)(q29),del(6)(q12),add(7)(p15), add(11)(p15),add(19)(p13),add(19) (q13), +r,inc[13]/46,XX[8] | No imbalances |
| 60/03-1209 ^a^ | S; hg | 76~81<4n>,XX,-X,-X,add(1)(p36)x2, del(2)(p16)x2,add(3)(p12)x2,add(3) (p13)x2,-4,-4,add(4)(p16)x2,der(6)t(3;6) (p21;q22)x2,t(8;17)(p23;q11)x2,i(9)(q10), -5,der(17)t(?6;17)(p21;q22)x2,-19,-19,-20, -20,-21,-21,-22,-22,+mar[15] | rev ish enh(1q23q31,2p13,2p21,2q21q36,3q21qter,4p11p15, 4q13q28,5p14p15,5q12q23,5q33q34,6p12,6q12q16,6q22q23, 8q13q24,9p12p21,9p21p24,9q13q21,11p14p15,11q14q22,12q15q21,13q21q32,20p12,20q),dim(X,1p32pter,3p13pter,6q25qter,8p21pter, 15q22qter,17p,17q11q21,19,21q21qter,22q) |
| 61/03-1211 | S | Culture failure | rev ish enh(Xp21pter,Xq13q27,1p31p33,1q21q24,1q31,2q22q31, 2q34,3p12p13,3p23,3q,4p,4q12q34,5p14p15,6p12,6q12q21, 7q31qter,8q21,8q22qter,9p13p24,9q21q22,11p14,11q13q23, 11q24q25,12p,12q21,13q21q22,20p12pter,21q21),dim(1p36, 2q13q21,5q13q31,5q33qter,6q22qter,7p12pter,8p21pter,10q22q24, 10q25q26,12q23qter,13q33qter,14q11q12,14q32,16p11p13,16q,17p,17q11q21,18q,19p13,19q13,21q22,22q),amp(3q24qter) |
| 62/04-86 | S; hg | 52~54,del(1)(q12),del(3)(p13),inc[9]/ 46,XX[26] | rev ish enh(Xq13qter,1p21p31,1q43qter,2p11p22,2q11q37,3p12,3q, 4p16,4q,6p12p21,6q12q24,7p11p15,7p15pter,7q11q35,8q,16p13, 19p13,19q13,21q21q22),dim(Xp,1p36,1q23,3p21p22,5q11q32,5q35,6q27,8p21,9q22qter,10p12,10q22qter,11q12q13,11q23qter, 12q23qter,14q22q24,14q31q32,15q13qter,16q,17p,17q24q25,19q13,20q12,22q12qter) |
| 63/04-349* | S; hg | 69~79,XX,del(X)(q22),del(1)(p13),add(4) (p14),+del(6)(q21),+del(7)(q22), -11, dup(11)(q13q25),der(11)add(11)(p15) dup(11)(q13q25),+add(12)(p13),der(19) (:19p13→19q13::11q13→11q23: :7q31→ 7qter),inc[cp10] | Not informative |
| 64/04-464 | S; hg | 61~73,XXX,+1,der(1)(1qter→1p34::1p11→1qter),add(2)(p11),del(2)(q32),der(3) add(3)(p21)add(3)(q29),-4,+5,i(6)(p10)x2, +7,add(7)(q36)x2,-8,del(11)(q22)x3, add(12)(q24),-14,-15,-16,add(17)(p11),-18, -19,-19,-20,-21,+5~15mar[22] | rev ish enh(1p13p31,1q,2q14q31,3p12p13,3q12q13,3q24qter,4q13, 5p12p15,5q11q23,5q32q34,6p,7p15,7p21,7q21q22,7q31,8q12qter, 11q14q22,12p,12q14q21,18q12q21),dim(Xp11,Xp21pter,Xq21qter, 1p33pter,2q37,3p14p22,6q25qter,7q34qter,8p12p23,9q22q31,9q34, 11q23q25,13q12q13,13q33qter,14q22qter,15q11q24,16q13qter, 17p13, 19p13,19q,21q22,22q11q13)amp(3q26) |
| 65/04-499 | S; hg | 46,XX[5] | rev ish enh(Xq12q21,Xq24qter,1p13p21,1q21q31,2p,2q11q12, 2q22q24,2q32q37,3q,4p12p15,4q12q22,4q33qter,5p13p15,6p11p21,6p22p25,6q12q21,7p11p22,7q11qter,8p12,8q11q24,10p12pter, 10q11q23,10q25,11p11p15,11q14q23,12p11p13,12q12,13q21qter, 14q22q23,14q31,15q15qter,20p12),dim(Xp21pter,1p22pter,2q14q21,2q31,3p14pter,4q31,5q11qter,6q22qter,9p21pter,12q14qter, 13q12q14,16q,17p,17q11q12,18q,19p13,19q13,21q21q22,22q), amp(1q24q31,2p22pter,3q26,7q31) |
| 66/04-715 | S; hg | Culture failure | Not informative |
| 67/04-760 ^a^ | S; hg | 46,XX[8] | rev ish enh(3p12p13,3q12q13,8q13q23,12p11p12),dim(6p21,6q25, 8p21p23,9q22qter,11q13,13q12q13,15q22q24,16q22q23,17p12p13, 17q12q21,22q11q13) |
| 68/04-972 | S; hg | 38,XX,del(1)(p32),inv(3)(p21q29),t(3;4) (p23;q21),-4,-6,-8,add(10)(p12),-11,-15, -15,-16,-17,del(18)(q21),del(19)(p13), der(19)t(?15;19)(q21;q13),-21,-22, +2mar[12]/70~78,idemx2[3] | No DNA available |
| 69/04-995* | S; hg | 50~59,dup(1)(q21q41),i(5)(p10),add(6) (q22),add(7)(q22),del(7)(q22),add(11) (p15),add(11)(q25),add(16)(q24),der(19) t(15;19)(q26;p13),add(19)(q13),inc[10] | rev ish enh(1p21p31,1q23q25,2q22q24,3q13,3q24q26,5p,5q31q33, 6p12p21,7q31q35,8q,11q14q22,12p11,12p12p13,12q14q21,18q12, 20p11p13),dim(Xp11p21,Xq12q13,Xq21q27,1p35p36,2q37,3p21, 6q25q26,8p21p23,9q33q34,10q25q26,12q23q24,13q12q14, 14q31q32,15q12q15,15q21q23,16p11p12,16q22q23,17p11p13, 17q11q25,18q21q23,19q13,21q22, 22q11q13) |
| 70/04-1019 | S; hg | culture failure | rev ish enh(1p31,1q21q25,3q26,4p16,4q12q21,8q12q13,8q23q24, 10q21q23,11p,11q12q14,11q23q24,12p,12q12q15,16p12p13, 17q24q25,19p13,19q13,20,22q11q13),dim(4q24qter,5p14pter, 5q14q21,8p12pter,13q12q32) |
| 71/04-1091 | S; hg | Culture failure | rev ish enh(3p12p14,4q26,6q12q13,6q15q16,8q23,13q21q31), dim(1p34pter,2q37,9q34,11q12q13,12q24,15q22,16p12p13, 17p11p13,17q11q21,17q24q25,19,20q11q13,21q22,22q) |
| 72/04-1096* | S; hg | 66~73,XXX,+der(1;6)(q10;p10),del(3) (p12),add(4)(q35),add(6)(q27),+del(7) (p13),add(14)(p11),der(19)(19qter→ 19p13::hsr19p13::22q12→22q13::6q16 →6qter),ider(19)(p13)hsr(19)(p13), inc[9] | Not informative |
| 73/04-1109 | S; hg | 89~93,XXXX,+1,i(11)(q10),add(13) (p11),add(16)(q24),add(19)(p13)x2, +20[4]/93~95,idem,+add(3)(p14)[4]/ 46,XX[22] | rev ish enh(1p13p34,1q,2p12p25,2q12q24,2q31q36,3,5p14p15, 5q11q23,5q31q33,8,11p11,11q13qter,20),dim(Xp21pter,7p12p21, 7q11q36,9p12p23,9q21qter,12p11p13,12q12q13,12q23q24,14q, 15q11,15q22q24,16,17p12p13,17q12q21,18p11,18q,19p13,19q13, 22q11q13) |
| 74/00-626 | S; lg | Culture failure | rev ish enh(1q24,2p,2q11q37,4q13,5q14,5q21,6q15q16,7p13p21, 7q21q35,8q,13q21q31),dim(Xq12,Xq13,1p31pter,5q23q32,9q22qter,10p12,10q23q24,10q26,11q12q13,12q23qter,13q12,14q31, 15q22q24,16p11p13,16q13qter,17p11p13,17q11q21,17q22q25, 18p11,18q21,19p13,19q,20q11q13,21q,22q11q13) |
| 75/00-736 | S; lg | Culture failure | rev ish enh(1q),dim(X,1p32p36,11q13,17p12p13, 17q12q21, 22q12q13),amp(1q31) |
| 76/01-435 | S; lg | 45~53,XX,+add(3)(q12)[cp2]/46,XX[39] | No DNA available |
| 77/01-882 | S; lg | 45,XX,-11[4]/45,X,-X[3]/46,XX[83] | No imbalances |
| 78/03-1020 | S; lg | 47,XX,+7[19] | rev ish enh(7) |
| 79/04-242 | S; lg | 46,XX[5] | Not informative |
| 80/04-414 | S; lg | 46,XX[17] | No DNA available |
| 81/62-99 | E | 40~44,XX,+add(1)(p31),del(1)(p32), add(2)(q32),add(3)(q29),del(3)(p21), +add(4)(q35),del(6)(q22),-7,-10,-11,-11, add(12)(q24),+14,add(14)(p11)x2,-19, der(19)t(11;19)(q21;p13),+1~4mar[cp10] | No DNA available |
| 82/00-577 | E | 41~43,XX,add(4)(p14),der(12)t(12;17) (p11;p11),?+del(12)(q10),-13,-15,-15, del(17)(p11),?der(22)t(15;22) (q13;q13)[17]/46,XX[12] | rev ish enh(1p12p21,1q21q23,1q32qter,3q24qter,6p12p22,6p24pter, 8q24,10q21qter,11p11p13,11q12q13,13q32qter,17q11q21, 17q23qter),dim(Xp21p22,3p12p14,4p12p16,4q12q34,6q16q23, 9q13q22,14q21q23) |
| 83/00-752 | E | 49~51,XX,del(6)(q15q23), +3~5mar[3]/ 88~95,idemx2[5] | No imbalances |
| 84/00-1168 | E | Culture failure | rev ish enh(1p13p22,1p31p34,1q23q32,2p11p24,2q14q24,2q31q36, 3q25q26,5p13,5p14p15,6p11p21,6p22p25,7p11p21,7q21,7q31q32, 8q12q24,10p,10q11,11p12p13,11q22qter,12p,12q12,13q14, 13q21q33,14q21,14q24,15q15q21,15q24q26,18q12,20),dim(Xp11, Xp21pter,Xq,1p36,3p12p26,3q21,5q11q32,5q34q35,6q25q26, 8p12p23,9p12p13,9q21qter,10q22,10q23q24,10q26,11q13, 12q23q24,16p11p13,16q21q24,17p11p13,17q11q22,17q23q25, 19p13,19q13,21q22,22q11q13),amp(3q26,12p) |
| 85/01-128 | E | Culture failure | rev ish enh(1q,8p,8q11q24,20q12q13),dim(1p,5q35,7p21p22,7q11, 7q35q36,9q33qter,10q24qter,11q13,12q23,12q24,14q31qter, 15q22q24,16p,16q23qter,17p11p13,17q24q25,19p,21q22,22q), amp(1q42q44) |
| 86/01-196 ^a^ | E | 46,XX[16] | rev ish enh(1q23q24,2q23q24,5q14,8q22,9p23,13q22),dim(1p32p36, 7q11,9q22,11q12q13,12p12,12q23q24,16p11p13,17p12p13,17q21, 19p13,19q13,22q11q13) |
| 87/01-987 | E | Culture failure | rev ish enh(Xp21,1q,2q22q24,3p12p14,3p21p25,3q12q26,5p, 5q11q31,5q32q35,8q,9p13p24,10q11q22,10q24q25,12q15q21,20p), dim(1p32pter,2p21p22,2p24,2q36q37,4p16,4q31qter,6p21p22,6q25, 7p11p12,7q11,7q21q22,7q31q36,9q22,9q31,9q33qter,10p12, 11q12q13,11q22q25,12q23q24,13q12,14q22q24,14q31q32, 15q22q24,17,19,20q,21q22,22q11q13) |
| 88/01-1117 | E | Culture failure | No DNA available |
| 89/01-1160 | E | 46,XX[89] | rev ish enh(1q21q42,2q22q24,3p12p14,3q12q13,5q14q22,6q15q16, 8q22q23,13q21q31,18q12),dim(1p32p36,4q34q35,6p21,6p22,6q25, 7q11,7q36,9q22q31,9q33q34,10q23,11q12q13,12p12,12p13, 12q23q24,14q23,14q31q32,15q22q23,16p11p13,16q13q24, 17p11p13,17q11q21,17q23q25,19p13,19q, 20q12,21q22,22q11q13) |
| 90/02-50 ^a^ | E | 37~38,inc[2]/46,XX[91] | rev ish enh(1p13p31,1q23q25,1q32q44,2p11pter,2q14,2q21q24,3p12, 3q12q13,3q21qter,5p,6p12,6p22,6q12q21,6q22,8q,10q23q26,11p13,11q14q23,11q24,12p,13q12,13q32q33,18p11,18q12,20p11p12, 20q13),dim(Xp21p22,Xq,1p33p36,3p14p22,3p24,4p13p15,4q, 5q12q14,5q21,5q23q35,6q24q26,7p21p22,8p12p23,9p12p21,9q, 10p11p13,12q23,12q24,13q21,14q22q32,15q11q23,15q26,16p11p13,16q21q23,17p,17q11q24,18q21qter,19p13,19q13,21q21q22, 22q11q13),amp(5p14) |
| 91/03-231 | E | Culture failure | rev ish enh(1q24,1q31,8q13q23),dim(1p34p36, 9q22qter,17q21) |
| 92/03-896 | E | 46,XX[4] | rev ish enh(1p13,1p31pter,1q21q23,2p11p12,2p13,2p14,2p22p25, 2q11q21,2q35q37,3q13q24,3q27qter,4p15pter,5p12pter,6p12p24, 6q25q27,7p12p15,7p21p22,7q11q21,7q21q31,7q31qter,9p12,9q, 11p11p13,11p14p15,11q12q14,11q22q24,12p13,12q12q14, 12q22q24,13q12q14,13q31qter,15q22q25,18q,19p13,19q13, 20p11p13,20q,22q11q13),dim(Xp11p22,Xq13q27,3p12p14,3p21p24,3p24p25,4q13qter, 5q12q33,8q13q23,10p11p14,10q11q25,10q26, 14q12q31,16q21q22,18p11,21q21q22) |
| 93/04-743 | E | 46,XX[3] | rev ish enh(Xq12,1p31p34,1q,2p13pter,3q25qter,6p,6q22q27, 7p11p12,7q11,8p11p12,8q11q22,8q24,11p11,11q12q14,12p11p13, 18p11,20p11,20q11q12),dim(1p36,6q13q21,8p21pter,11p14p15, 11q22q23,11q23qter, 21q21q22),amp(3q25q29, 6p21p22) |
| 94/04-1051 | E | 42,XX[42] | rev ish enh(3p12,3q12q13,5q14,5q21,6p11p12,6q12q21,6q21q23, 9p24,13q21q31,21q21),dim(3p21,7q35,9q34,10q25q26,11q13,12p13,12q23q24,14q31,15q22q24,16p11p13,16q22q24,17p12p13, 17q11q21,17q22q25,19p13,19q13,20q13,22q) |
| 95/04-1141 | E | 46,XX[4] | rev ish enh(Xp22,Xq12q21,Xq21q23,Xq24,Xq25,Xq26q27,1q21, 1q22q42,2p12p13,2p14p16,2p21,2p23p24,2q13q21,2q22q24, 2q24q33,2q33q36,3p12p21,3p21p24,3q12q27,7p13p21,7q21, 7q22q31,7q31q32,7q33q35,8p12p22,8q12q24,10p11p13,10q11q26, 13q13,13q22,16p12p13,20p11p12,20q12q13),dim(1p13pter, 6q12qter,15q12q26,18p11,18q12qter,19p13,19q13,22q) |
| 96/00-20 | CC | 75,der(1)del(1)(p34)del(1)(q32),inc[2]/ 131~146,idemx2[2]/46,XX[12] | rev ish enh(1p34p35,9q33q34,10q22,19p13,19q13, 20q11,22q11q12) |
| 97/01-357 | CC | 52~82<3n>,XXX,+1,+del(2)(q32),-4,-9, -11,+13,der(13;14)(q10;q10)x2,add(14) (p10),+add(14) (p10),-15,+16,-20,+21,+22, +2mar[cp10]/ 46,XX[3] | rev ish enh(1p13,1p21p31,1q23q32,2p11p25,2q12q32,3p12p25, 3q12q26,5p13pter,5q11q23,5q31q34,7p13p22,7q21,7q22q34,8p11, 8q,9p13p24,10,14q24q31,17q22q23,20q,21q,22q11q13),dim(Xp, Xq13,4p,4q12, 4q21q33,6p21pter,6q25q26,9q21qter,11q13,11q21, 11q23,12p12,12p13,12q12q13,12q23qter,13q,14q11q12,15q11q21, 15q22q23,16p11p13,17p,18p,18q21q22,19p13,19q,20p11pter), amp(8q) |
| 98/01-461 | CC | Culture failure | No DNA available |
| 99/03-60 | CC | 46,XX[8] | rev ish enh(1q23q31,1q31q44,2p13,2p21p24,2q11q32,3q13q24, 5p13p15,5q12q13,5q14q23,5q32q35,7p14p21,8q12qter,10q11q21, 10q22q23,10q23q25,12p,12q12q21,17q11q23,19q13),dim(Xq21q28,4q13q24,6p21p22,9q22q31,13q,16q) |
| 100/03-268 | CC | 63~85<3n>,del(X)(q22),add(X)(q28),+1, i(1)(q10)x2,add(1)(p34),-2,del(3)(q21),-4, add(5)(p11),-6,add(6)(p25),+7,i(8)(q10),-10, add(11)(q22),i(11)(q10),add(19) (p13)x2,-21, inc[8]/46,XX[2] | rev ish enh(1q21qter,2p12p21,2p23p24,2q14q34,3p,3q12q26,5p, 7p11p21,7q11q36,8p12pter,8q,10q21,12p11p13,12q12,14q21, 16q13q21,20q),dim(1p11p22,1p32p36,4p15p16,4q21q28,4q28q31, 4q32qter,5q32qter,6p11p21,6q12,6q13q15,6q16qter,9q32qter, 10p12pter,11p,11q12q13,12q23qter,13q12q21,14q23q24,14q24q32, 15q22q26,16p11p13,17p,17q11,17q12,17q21,17q24qter,19,21q22, 22q11q12,22q12q13),amp(8q13q21,8q22q23) |
| 101/03-371* | CC | 79~81<3n>,XXX,+add(1)(p31), der(1) t(1;1)(p22;q21),-2,-2,-2,+add(5)(p13), idic(6)(q22),+7,i(8)(q10)x2,+12,ins(12;?) (q12;?)x2,+der(18)(18pter→18q12::?:: 1p12→1pter)x2,add(19)(p13),+20,-21, +6~9mar[10] | rev ish enh(Xp11,Xq12q13,Xq21,Xq21q22,Xq23,Xq24q25,Xq26q27, 1p31p34,1p36,1q,2p16p22,2p24,2q11q22,5,7p11p13,7p14p21,7q11, 7q21qter,8p11,8q, 9p21p23,9q22q32,10q11q26,12p11p13,12q14q21, 12q22q24,14q11q13,14q31q32,17q,19q,20p11p12,20q12qter, 22q11q13),dim(2q36qter,4p14,4q13q21,4q25q27,4q28,4q31, 6p21p22,6q22q24,6q24q25,6q25q27,8p12pter,13q21,13q22q31, 13q32,16p13,16q23qter) |
| 102/02-416 ^a^ | CC | 48,XX,+2mar[3]/46,XX[42] | No imbalances |
| 103/04-1060 | CC | Culture failure | Not informative |
| 104/00-780 | M | Culture failure | rev ish enh(7q),dim(1p32p35,9q34,11q13,12q24,17q12q21, 17q24q25,19p13,19q13,22q11q13),amp(7q31) |
| 105/01-582 | M | 84~90,XXXX,add(7)(q32)x2,add(13) (p13)x2,add(15)(p13),-16,i(17)(q10), add(22)(p13)[15] | rev ish enh(1q24,2q22q24,2q32,3p12p14,3q12q13,4p15,4q13,5p14, 5q14q22,6q13q16,7q22q32,7q34,8q21,8q22q23,8q24,9p23p24, 11p14p15,13q21q32,17q,18q12,21q21),dim(1p32pter,5q31,7p13, 7q11,7q36,9q22q31,11q12q13,12p12,12q23,12q24,14q31q32, 15q13q14,15q22,15q23q24,15q26,16p11p13,17p,18p11,19p13,19q, 20q11q13,21q22,22q) |
| 106/01-700 | M | 46,XX[116] | rev ish enh(8q23),dim(1p34p35,7q11,17p12p13,19p13,19q13, 22q11q12) |
| 107/01-667 | E+M | 46,XX,add(8)(q24)[5]/46,XX[88] | No imbalances |
| 108/02-278 ^a^ | E+M | 46,XX[91] | No imbalances |
| 109/04-1261 | E+M | Culture failure | rev ish enh(1q,2p11p16,2p21p22,2p23pter,2q11q12,2q14q37,10p11, 11p11,16q12q13),dim(1p13pter,6q12qter,9p,9q21qter,22q11q12) |
| 110/02-524 | U | 46,XX[26] | rev ish enh(1p31p34,3q12q28,4q21q25,6p22,8q11q22,8q22q24, 11p12p14,11q13q14,12p,12q12q24,14q11q12,14q22qter,18p, 18q11q21,19p13,19q13,20p11p12,20q11q12),dim(Xp22,2p24p25, 4q33q34,5q35,9q33,13q,15q11q15,15q22q24,17p12p13,21q22, 22q13),amp(12q13) |

S serous; E endometrioid; M mucinous; U undifferentiated; CC clear-cell; hg high grade; lg low-grade.

*Tumors published in Micci et al., 2009

^a^Tumors from patients who had received neoadjuvant therapy.
